# Contributions of Ca_V_1.3 Channels to Ca^2+^ Current and Ca^2+^-Activated BK Current in the Suprachiasmatic Nucleus

**DOI:** 10.3389/fphys.2021.737291

**Published:** 2021-09-28

**Authors:** Beth A. McNally, Amber E. Plante, Andrea L. Meredith

**Affiliations:** Department of Physiology, University of Maryland School of Medicine, Baltimore, MD, United States

**Keywords:** CACNA1D, KCNMA1, BK channel, L-type Ca^2+^ channel, circadian rhythm, Ca^2+^-activated K^+^ channel

## Abstract

Daily regulation of Ca^2+^**–** and voltage-activated BK K^+^ channel activity is required for action potential rhythmicity in the suprachiasmatic nucleus (SCN) of the hypothalamus, the brain's circadian clock. In SCN neurons, BK activation is dependent upon multiple types of Ca^2+^ channels in a circadian manner. Daytime BK current predominantly requires Ca^2+^ influx through L-type Ca^2+^ channels (LTCCs), a time when BK channels are closely coupled with their Ca^2+^ source. Here we show that daytime BK current is resistant to the Ca^2+^ chelator BAPTA. However, at night when LTCCs contribute little to BK activation, BK current decreases by a third in BAPTA compared to control EGTA conditions. In phase with this time-of-day specific effect on BK current activation, LTCC current is larger during the day. The specific Ca^2+^ channel subtypes underlying the LTCC current in SCN, as well as the subtypes contributing the Ca^2+^ influx relevant for BK current activation, have not been identified. SCN neurons express two LTCC subtypes, Ca_V_1.2 and Ca_V_1.3. While a role for Ca_V_1.2 channels has been identified during the night, Ca_V_1.3 channel modulation has also been suggested to contribute to daytime SCN action potential activity, as well as subthreshold Ca^2+^ oscillations. Here we characterize the role of Ca_V_1.3 channels in LTCC and BK current activation in SCN neurons using a global deletion of CACNA1D in mouse (Ca_V_1.3 KO). Ca_V_1.3 KO SCN neurons had a 50% reduction in the daytime LTCC current, but not total Ca^2+^ current, with no difference in Ca^2+^ current levels at night. During the day, Ca_V_1.3 KO neurons exhibited oscillations in membrane potential, and most neurons, although not all, also had BK currents. Changes in BK current activation were only detectable at the highest voltage tested. These data show that while Ca_V_1.3 channels contribute to the daytime Ca^2+^ current, this does not translate into a major effect on the daytime BK current. These data suggest that BK current activation does not absolutely require Ca_V_1.3 channels and may therefore also depend on other LTCC subtypes, such as Ca_V_1.2.

## Introduction

The suprachiasmatic nucleus (SCN) is the central circadian clock of the brain in mammals (Hastings et al., 2018; Harvey et al., 2020). Membrane signaling *via* ion channels regulates the daily patterning of action potential activity and neurotransmitter release, which is critical for the expression of circadian behavioral rhythms. Calcium is an important regulator of SCN signaling (Ikeda, 2004; Hastings et al., 2018; McNally et al., 2020), and SCN neurons have several different Ca^2+^ influx pathways, including voltage-gated Ca^2+^ channels (Pennartz et al., 2002; Cloues and Sather, 2003; Harvey et al., 2020; McNally et al., 2020). Of the channels involved in SCN signaling, L-type Ca^2+^ channels (LTCCs) are abundantly expressed (Nahm et al., 2005; Harvey et al., 2020).

LTCC currents are generated by the Ca_V_1 family of Ca^2+^ channel subtypes (Lipscombe et al., 2004; Striessnig et al., 2006). Ca_V_1.1 is the skeletal muscle LTCC, while Ca_V_1.2 (α1C, CACNA1C) and Ca_V_1.3 (α1D, CACNA1D) are the predominant subtypes in neurons and are sensitive to inhibition by dihydropyridines such as nimodipine (Lipscombe et al., 2004; Striessnig et al., 2006). Ca_V_1.2 plays a wide variety of roles in neuronal excitability and cell signaling, regulating action potential activity, secretion, and transcription (Striessnig et al., 2006), but global genetic deletion of Ca_V_1.2 is lethal due to cardiac dysfunction (Seisenberger et al., 2000). Ca_V_1.3 is also expressed in variety of neurons and neuroendocrine cells, often co-expressed with Ca_V_1.2, but differs from Ca_V_1.2 in its activation at lower voltages (Striessnig et al., 2006). A global deletion of Ca_V_1.3 channels is viable and results in deafness and cardiac arrhythmias (Platzer et al., 2000; Zhang et al., 2005). SCN neurons express both Ca_V_1.2 and Ca_V_1.3 channels (Nahm et al., 2005; Huang et al., 2012; Kim et al., 2015; Cheng et al., 2018).

SCN LTCC current is diurnally modulated, with higher current during the day that decreases at night (Pennartz et al., 2002; Whitt et al., 2018; McNally et al., 2020). Correlated with the change in current magnitude, inhibition of LTCC current with nimodipine decreases SCN firing selectively during the day (Pennartz et al., 2002; Whitt et al., 2018; McNally et al., 2020). One mechanism for the effect on SCN firing is *via* LTCC-dependent activation of Ca^2+^-activated BK K^+^ channels (Plante et al., 2021). During the day, LTCCs are responsible for the majority of BK current activation (Jackson et al., 2004; Whitt et al., 2018; Plante et al., 2021). However, at night, their role is reduced, and BK current activation relies primarily on release of Ca^2+^ from intracellular stores (Whitt et al., 2018; Plante et al., 2021). Either Ca_V_1.2 or Ca_V_1.3 could contribute to the LTCC current that drives daytime activation of the BK current, as both channel subtypes are known to activate BK currents produced by BK channel variants from SCN (Plante et al., 2021) or within other excitable cells (Roberts et al., 1990; Wisgirda and Dryer, 1994; Prakriya and Lingle, 1999; Sun et al., 2003; Grunnet and Kaufmann, 2004; Berkefeld et al., 2006; Marcantoni et al., 2010; Hou et al., 2016; Bellono et al., 2017; Vivas et al., 2017; Plante et al., 2021). Little is known about the specific role of Ca_V_1.2 on SCN firing, but Ca_V_1.2 expression is higher at night and mouse knockouts display altered nighttime phase-shifting behavior (Schmutz et al., 2014). In contrast, Ca_V_1.3 channels have been proposed to regulate firing rate, Ca^2+^ oscillations, and histamine signaling during the day (Huang et al., 2012; Kim et al., 2015, 2016). Current pharmacological tools cannot effectively distinguish between these LTCC subtypes, necessitating the use of transgenic mouse lines to address the relative contributions of these channel subtypes to neuronal excitability.

In this study, we focused on investigating a role for Ca_V_1.3 channels in BK current activation, based on their proposed roles in SCN during the day and their regulation of spontaneous firing in other cell types *via* BK channel activation (Vandael et al., 2010; Bellono et al., 2017). Using a Ca_V_1.3 knockout mouse line (Ca_V_1.3 KO, also called α1D^−/−^, CACNA1D^−/−^)(Platzer et al., 2000), whole-cell Ca^2+^ and BK currents were recorded from day and night SCN neurons in acute brain slices. The results reveal that Ca_V_1.3 channels contribute to the daytime LTCC current in SCN neurons, as well as BK current activation.

## Materials and Methods

### Mice

Experimental mice were 3–6 week old male and female wildtype (WT) C57BL/6J and Ca_V_1.3 WT and KO littermates produced from heterozygous Ca_V_1.3 breeding pairs on a mixed C57BL6:FVB background (Platzer et al., 2000). Tail or ear tissue samples were genotyped using “Cacna1d-1 WT” and neomycin probes in real-time PCR reactions at a commercial vendor (Transnetyx, Cordova, TN). All mice were group housed from birth on a standard 12:12 h light–dark cycle (for day timepoints) or a reverse 12:12 h light–dark cycle (night timepoints). All procedures involving mice were conducted in accordance with the University of Maryland School of Medicine Animal Care and Use Guidelines and approved by the Institutional Animal Care and Use Committee.

### Acute SCN Slice Preparation

Mice were sacrificed at zeitgeber time (ZT) 1–3 h for day, or ZT 14–16 h for night, experiments. Brains were rapidly removed and placed into ice-cold sucrose-substituted saline containing (in mM): 1.2 MgSO_4_, 26 NaHCO_3_, 1.25 Na_2_HPO_4_, 3.5 KCl, 3.8 MgCl_2_, 10 glucose and 200 sucrose. Coronal slices (300 μm) were cut using a VT1000S vibratome (Leica Microsystems, Wetzlar, Germany) at 3–4°C. Slices containing SCN were incubated 1–2 h at 25°C in oxygenated artificial cerebrospinal fluid (ACSF) containing (in mM): 125 NaCl, 1.2 MgSO_4_, 26 NaHCO_3_, 1.25 Na_2_HPO_4_, 3.5 KCl, 2.5 CaCl_2_ and 10 D-glucose (300–305 mOsm/kg). Slices were transferred to the recording chamber and perfused *via* gravity flow at 1–2 ml min^−1^ with oxygenated ACSF.

### Electrophysiological Recordings

Recordings were performed at the peak (ZT 4–8 h) and nadir (ZT 17–21 h) of the circadian rhythm in spontaneous action potential firing, corresponding to the “day” and “night” timepoints, respectively. Neurons within the center of the SCN were identified in whole-cell current-clamp mode by spontaneous action potential firing or firing following injection of 5–20 pA of current for silent neurons.

Macroscopic BK and Ca^2+^ currents were recorded in whole-cell voltage-clamp mode at 25°C as described previously (Whitt et al., 2018; McNally et al., 2020). For BK currents, electrodes (4–7 MΩ) were filled with intracellular solution (in mM): 123 K-methanesulfonate, 9 NaCl, 0.9 EGTA, 9 HEPES, 14 Tris-phosphocreatine, 2 Mg-ATP, 0.3 Tris-GTP, and 2 Na_2_-ATP, pH 7.3 (310–315 mOsm/kg). BAPTA (5 mM) was substituted for EGTA (0.9 mM) in some internal solutions as specified in figure legends. The bath ACSF was composed of (in mM): 125 NaCl, 1.2 MgSO_4_, 26 NaHCO_3_, 1.25 Na_2_HPO_4_, 3.5 KCl, 2.5 CaCl_2_ and 10 D-glucose (300–305 mOsm/kg). For Ca^2+^ currents, the internal solution was (in mM): 115 cesium gluconate, 10 tetraethylammonium chloride, 10 HEPES, 0.5 EGTA, 2 MgCl_2_, 20 sodium phosphocreatine, 2 Na_2_ATP, and 0.3 Na_3_ GTP (pH 7.3, 310–315 mOsm/kg). The bath ACSF was composed of (in mM): 68 NaCl, 3.5 KCl, 1 NaH_2_PO_4_, 26.2 NaHCO_3_, 1.3 MgSO_4_, 2.5 CaCl_2_, 10 D-glucose, 60 tetraethylammonium chloride, and 3 CsCl (300–305 mOsm/kg).

Total voltage-activated BK and Ca^2+^ currents were recorded in 1 μM tetrodotoxin (TTX) before and after focal perfusion (4-min wash-on) of the selective inhibitors paxilline (BK channels) or nimodipine (L-type Ca^2+^channels), respectively. A minimum 10–15-min wash-out period of focally and bath perfused ACSF was performed before recording from the next cell. BK and L-type Ca^2+^ currents were isolated by subtracting currents elicited in the presence of their respective inhibitors, Nimodipine and Paxilline respectively, from total baseline currents. Three currents were averaged per cell and normalized to cell capacitance (range of 5–10 pF). R_a_ was <25 MΩ with less than ± 5% change (on average ~15 MΩ). R_s_ was compensated at 60%. BK currents were elicited from a holding potential of −90 mV, stepping from −110 to 90 mV for 150 ms in 20-mV increments. Ca^2+^ currents were elicited from a holding potential of −90 mV, stepping from −90 to 50 mV for 150 ms in 10-mV increments. Only cells with a total current size of >100 pA were used in experiments. Voltage values were adjusted for the liquid junction potential (9 mV). Currents were *post hoc* filtered at 1 kHz.

### Pharmacology

Drugs used in these experiments were: L-type Ca^2+^ channel inhibitor nimodipine (Nim, 10 μM, Alomone Labs, Jerusalem, Israel, #N-150), Ryanodine Receptor inhibitor dantroline (Dan, 10 μM, Sigma, #D9175), BK current inhibitor Paxilline (Pax, 10 μM, Alomone Labs, Jerusalem, Israel, #P-450) and Sodium channel inhibitor tetrodotoxin (TTX, 1 μM, Alomone Labs, Jerusalem, Israel, #T-550). All drugs were dissolved in DMSO, except TTX, which was dissolved in water. Drugs were focally perfused to the bath at a flow rate of 1 ml min^−1^ by a computer-controlled pressurized perfusion system (ValveLink 8.2; Automate Scientific, Berkeley, CA, USA) at the concentrations indicated from 1,000× stocks.

### Membrane Potential Oscillations

In whole-cell current-clamp mode, membrane potential oscillations were recorded in TTX (1 μM) using the same solutions as BK current recordings (McNally et al., 2020). Cells with resting membrane potentials between −30 and 65 mV were included in the dataset. Data were acquired in 10-s sweeps at the cell resting membrane potential (spontaneous oscillations) and during a series of holding potentials stepping from −60 to 0 mV, in 10 mV increments (voltage-dependent oscillations). Oscillations were defined as at least a 5-mV change in membrane potential, with a frequency of 0.2 Hz. Representative traces in figures were at 2 kHz.

### Data Analysis and Statistics

BK and Ca^2+^ current-voltage plots were constructed from peak current level at each voltage step. Data are reported as group mean ± SEM. Numbers reported in figure legends are the number of neurons recorded, with 1–6 neurons per animal (one SCN slice per animal). Data for each condition was derived from a minimum of two animals. Statistical significance was determined at *P* < 0.05 using Prism v7 (GraphPad Software, Inc) using unpaired Welch's *t* tests, one-way ANOVA with Bonferroni's *post hoc* test, and Fisher's exact test for categorical data as indicated in results and figure legends. Representative current traces in figures were filtered at 500 Hz.

## Results

### Effect of Ca^2+^ Buffering on Day and Night BK Current Activation

L-type Ca^2+^ current is larger during the day compared to night in SCN neurons (Pennartz et al., 2002; Whitt et al., 2018; McNally et al., 2020). In conjunction with the diurnal modulation of LTCC current, BK current is predominantly dependent upon LTCCs during the day. However, LTCCs do not significantly contribute to BK current activation at night, suggesting the coupling between BK channels and their Ca^2+^ channel sources could differ between day and night (Whitt et al., 2018). To examine this idea, C57BL6 WT BK currents were recorded in two Ca^2+^ buffering conditions. As a control, BK currents were first recorded under standard whole-cell voltage-clamp conditions with 0.9 mM EGTA in the internal solution, conditions which permit the endogenous Ca^2+^ sources to contribute to BK current activation (Fakler and Adelman, 2008; Whitt et al., 2018; Plante et al., 2021). These BK currents recorded with 0.9 mM EGTA were then compared to BK currents recorded with internal solution containing 5 mM BAPTA, a Ca^2+^ chelator with fast kinetics that disrupts the functional coupling of BK channels located >10–20 nm from their Ca^2+^ source (Berkefeld et al., 2006; Fakler and Adelman, 2008; Cox, 2014). If the coupling between BK channels and their Ca^2+^ channel sources differs between day and night, BAPTA would be expected to reduce BK current below levels recorded in the EGTA control conditions.

**Figure 1:**
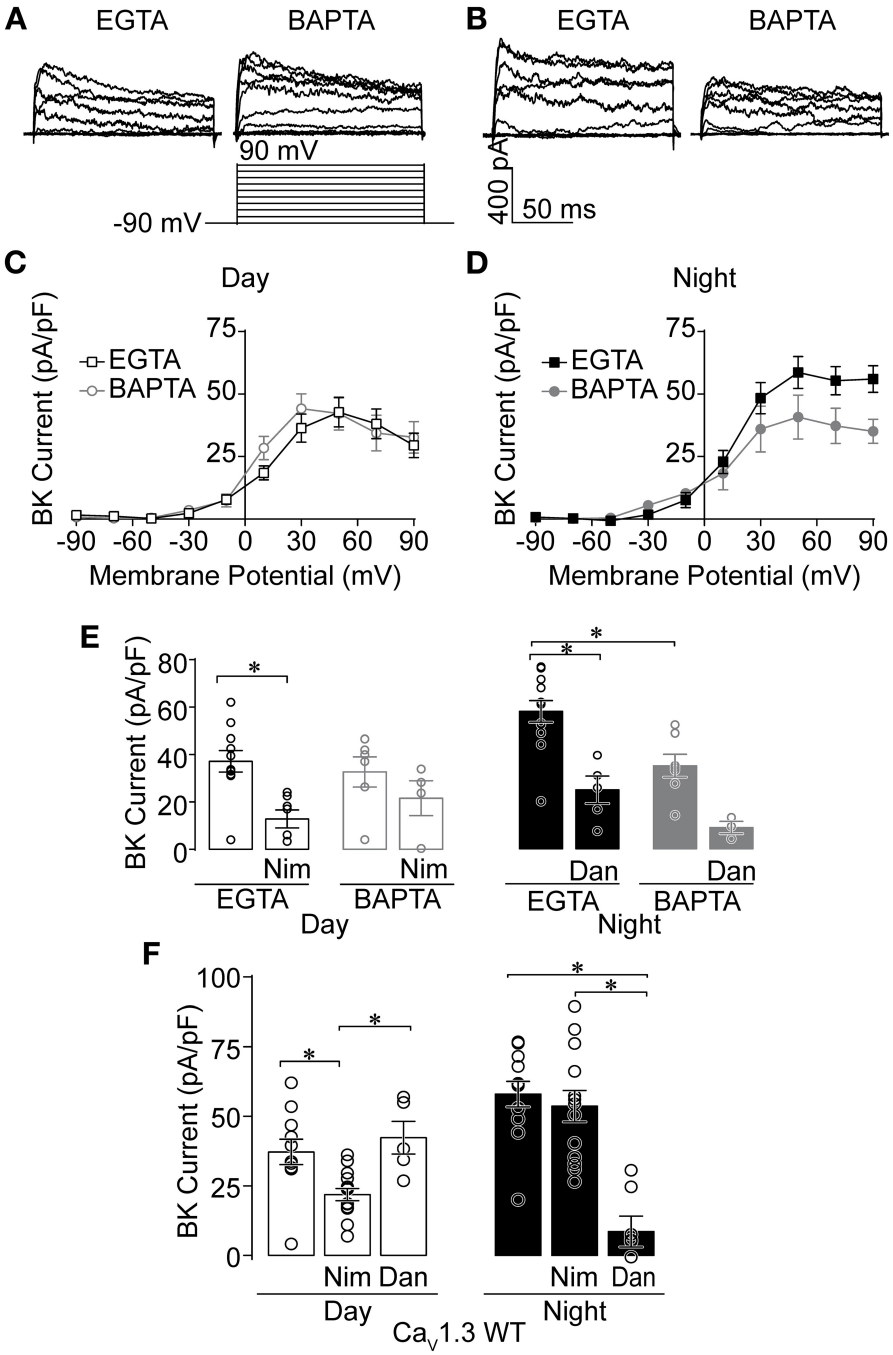
Effects of Ca^2+^ chelators and Ca^2+^ channel inhibitors on BK currents recorded from SCN neurons during the day and night. Paxilline-sensitive macroscopic BK currents were recorded from C57BL6 WT **(A–E)** and Ca_V_1.3 WT **(F)** SCNs. All intracellular solutions in this study were made with 0.9 mM EGTA, except where 5 mM BAPTA was substituted **(A–E)**. Currents were elicited from a holding potential of −90 mV by 150-ms voltage steps from −110 to +90 mV in +20-mV increments. **(A,B)** Representative BK currents from −90 to +90 mV are shown from day **(A)** and night **(B)** SCN neurons. **(C,D)** Current-voltage plot comparing BK current density recorded in either control EGTA or BAPTA during the day **(C)** and night **(D)**. **(E)** Summary of BK current density at +90 mV recorded with control EGTA or BAPTA with Ca^2+^ channel inhibitors 10 μM nimodipine (Nim) during the day, or 10 μM dantrolene (Dan) at night. In EGTA, Nim decreased BK currents compared to controls (*P* = 0.0008), with no significant difference in BAPTA. At night, BK currents were decreased with BAPTA compared to control EGTA conditions (*P* < 0.0001). Then Dan decreased BK currents in control EGTA (*P* = 0.001), but BAPTA was not significant. **(F)** Ca^2+^ channel inhibitor sensitivity in Ca_V_1.3 WT SCN neurons. Summary of BK current density at +90 mV recorded with EGTA in control (no drug) conditions, or in the presence of Nim or Dan in day and night. Ca_V_1.3 WT BK currents were decreased in Nim during the day compared to controls (*P* = 0.01), but not Dan. At night, Ca_V_1.3 WT BK currents were decreased in Dan compared to control (*P* = 0.008) and Nim (*P* < 0.0001), but Nim and control were not different. *P < 0.05, One-way ANOVA with Bonferroni's *post hoc* test between all conditions within day or night. N's represent individual cells recorded from C57BL6 WT (EGTA, BAPTA) day: control (11 neurons, three slices; six neurons, two slices); Nim (seven neurons, two slices; four neurons, one slice) and night: control (12 neurons, three slices; seven neurons, two slices); Dan (five neurons, two slices; three neurons, one slice). Ca_V_1.3 WT (day, night): control (11 neurons, six slices; 12 neurons, three slices), Nim (14 neurons, four slices; 13 neurons, four slices), and Dan (five neurons, two slices; seven neurons, two slices). Data are mean ± SEM.

Daytime BK currents were not reduced in BAPTA compared to EGTA (Figures 1A,C,E), suggesting tight functional coupling and close spatial localization between BK channels and their daytime Ca^2+^ source. However, in contrast to daytime, at night BK current was reduced ~37% in BAPTA (Figures 1B,D,E). This suggests that some BK channels do not remain closely localized with their Ca^2+^ source at night. This change in BAPTA sensitivity was correlated with a change in the Ca^2+^ source that activates BK channels (Whitt et al., 2018). During the day, LTCCs are responsible for the majority of BK current activation. At night BK activation relies primarily on release of Ca^2+^ from intracellular stores through RyRs. This differential activation can be probed with inhibitors for these Ca^2+^ sources.

Consistent with previous studies, daytime BK current was sensitive to inhibition by nimodipine, a selective inhibitor of LTCCs, in control EGTA (Figure 1E) (Whitt et al., 2018). In the presence of BAPTA, the reduction in BK current was not statistically significant (Figure 1E). At night, BK current in control EGTA was sensitive to inhibition by dantrolene, a selective inhibitor of RyR Ca^2+^ release from intracellular stores. In BAPTA, a similar result to daytime recordings was observed, in that this condition also lacked significance (Figure 1E). Thus, taken together, the inhibitor sensitivity in BAPTA still leaves open whether co-localized BK-Ca^2+^ channel complexes contain either LTCCs during the day or RyRs at night. During the day, part of the lack of a definitive effect could be due to the potential for multiple Ca_V_1 isoforms (Ca_V_1.2 and Ca_V_1.3) to contribute to the Ca^2+^ current in SCN neurons (Nahm et al., 2005; Huang et al., 2012; Kim et al., 2015; Cheng et al., 2018).

Given the influence of Ca_V_1.3 channels on firing rate and membrane oscillations during the day (Huang et al., 2012), and BK's ability to partner with Ca_V_1.3 without requiring other proteins in heterologous cells (Vivas et al., 2017; Plante et al., 2021), we first examined the specific contribution of Ca_V_1.3 to BK current activation by recording from Ca_V_1.3 KO SCNs (Platzer et al., 2000). Ca_V_1.3 KO mice were obtained on a C57BL6:FVB background, and Ca_V_1.3 WT littermates on this mixed strain background were used as controls. First, the changeover in Ca^2+^ source was verified in this strain from recordings in Ca_V_1.3 WT SCNs. As with inbred C57BL6 SCN neurons (EGTA recordings, Figure 1E) (Whitt et al., 2018), Ca_V_1.3 WT daytime BK currents were sensitive to inhibition by nimodipine, while dantrolene had a negligible effect (Figure 1F). At night, BK currents became nimodipine-insensitive, but dantrolene-sensitive (Figure 1F). This demonstrates that daily changeover in BK channel activation by its Ca^2+^ source occurs on the mixed strain background harboring the Ca_V_1.3 transgene.

### Ca^2+^ Current in Ca_V_1.3 KO SCNs

**Figure 2:**
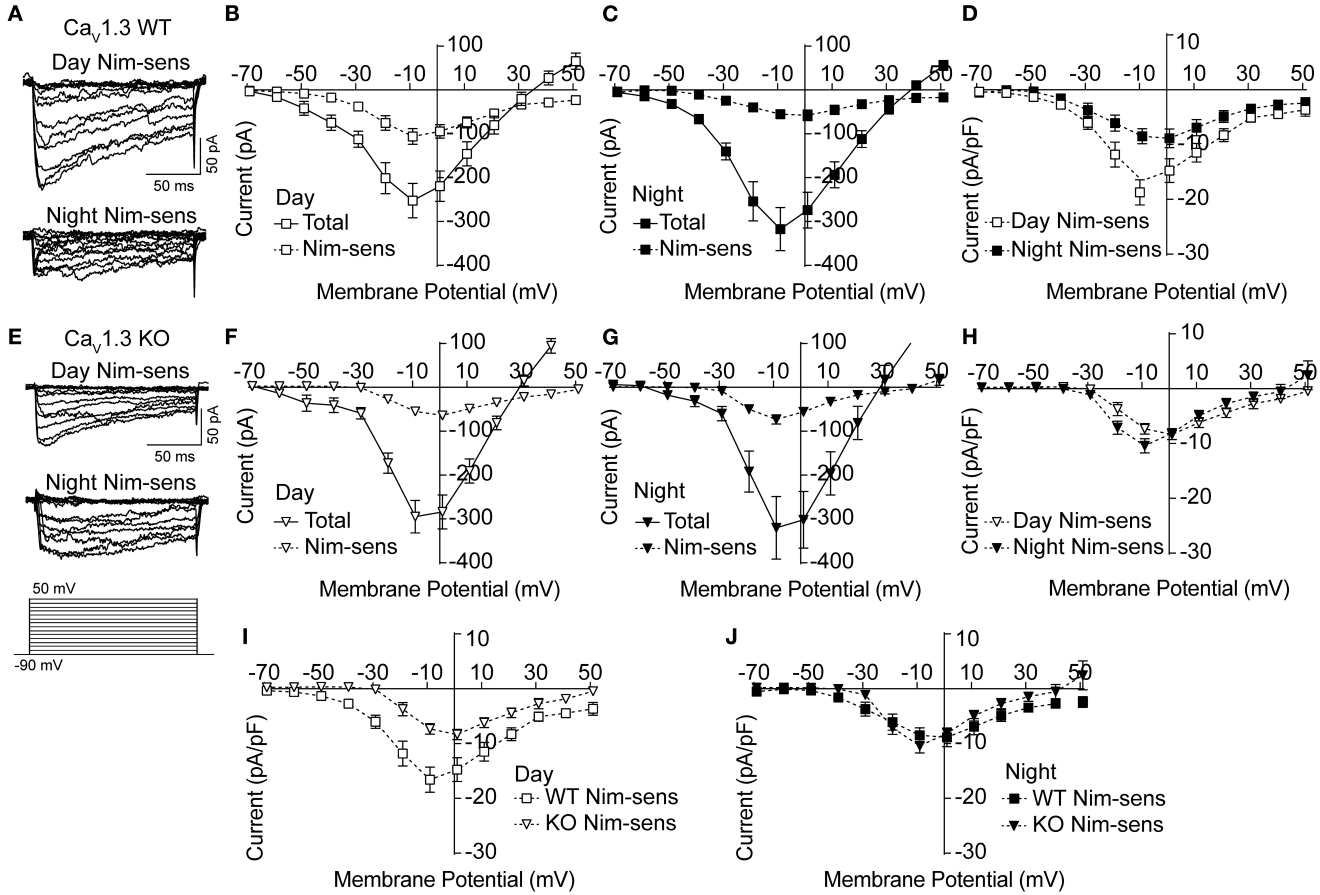
Nimodipine-sensitive Ca^2+^ currents from Ca_V_1.3 WT and Ca_V_1.3 KO SCN during the day and night. Macroscopic Ca^2+^ currents were elicited from a holding potential of −90 mV by 150-ms voltage steps from −90 to +50 mV in +10-mV increments. Nimodipine-sensitive LTCC currents were isolated by subtracting currents obtained in 10 μM nimodipine from the total cell current. **(A,E)** Representative nimodipine-sensitive (Nim-sens) current traces from Ca_V_1.3 WT **(A)** and Ca_V_1.3 KO **(E)** neurons during the day (top current traces) and night (bottom). **(B,C)** Current-voltage plot for Ca_V_1.3 WT Ca^2+^ currents before nimodipine (total) and the nimodipine-sensitive (Nim-sens) current from day **(B)** and night **(C)** neurons. **(D)** Comparison of Ca_V_1.3 WT nimodipine-sensitive normalized current density between day and night. Ca_V_1.3 WT nimodipine-sensitive currents were larger during the day (at −10 mV) compared to night (at 0 mV) (*P* = 0.01). **(E)** Representative nimodipine-sensitive currents from Ca_V_1.3 KO neurons during the day (top current traces) and night (bottom). **(F,G)** Current-voltage plot of Ca_V_1.3 KO total and nimodipine-sensitive currents from day **(F)** and night **(G)** neurons. **(H)** Comparison of Ca_V_1.3 KO nimodipine-sensitive normalized current density between day and night. Ca_V_1.3 KO nimodipine-sensitive currents were not different between day (at 0 mV) and night (at −10 mV) (*P* = 0.2). **(I,J)** Comparisons of nimodipine-sensitive current densities from Ca_V_1.3 WT and Ca_V_1.3 KO SCN during the day **(I)** and night **(J)**. Ca_V_1.3 KO nimodipine-sensitive currents were smaller than Ca_V_1.3 WT currents during the day (*P* = 0.009) but not at night (*P* = 0.5). *P* < 0.05, unpaired Welch's *t* tests. N's represent individual cells recorded from Ca_V_1.3 WT (eight neurons, three slices day; 10 neurons, four slices night) and Ca_V_1.3 KO (12 neurons, two slices day; nine neurons, one slice night). Data are mean ± SEM.

Next, the Ca^2+^ currents were characterized in Ca_V_1.3 WT and KO SCNs to determine if loss of Ca_V_1.3 channels results in decreased Ca^2+^ current (Figure 2). Ca^2+^ currents (Figures 2A,E) were recorded using the voltage protocol shown in Figure 2E. In daytime Ca_V_1.3 WT SCN neurons, the peak nimodipine-sensitive current is 42% of the total current. At night, this decreases to 16% (Figures 2B,C). This differential contribution generated a day vs. night difference in the nimodipine-sensitive LTCC current (Figure 2D) that is consistent with previous studies on other strain backgrounds (Pennartz et al., 2002; Whitt et al., 2018; McNally et al., 2020).

In the absence of Ca_V_1.3 channels, there was no change in the total Ca^2+^ current in the Ca_V_1.3 KO compared to WT (at −10 mV, *P* = 0.59 during the day and *P* = 0.76 at night, respectively; unpaired *t*-test) (Figures 2F,G). However, the peak nimodipine-sensitive current was reduced during the day in Ca_V_1.3 KO cells compared to WT (Figure 2I), comprising only 23% of the total Ca^2+^ current (Figures 2E,F). In addition, the peak of the daytime current-voltage relationship for the nimodipine-sensitive current also shifts from −10 mV (Ca_V_1.3 WT) to 0 mV (Ca_V_1.3 KO), consistent with loss of the low voltage activating Ca_V_1.3 channels (Figure 2I). Together these changes in the voltage-dependence and current magnitude identify that Ca_V_1.3 channels contribute to the daytime LTCC current in SCN neurons.

At night, the contribution of the nimodipine-sensitive current to the total Ca^2+^ current in Ca_V_1.3 KO neurons was similar to daytime contribution (22%, Figures 2G,H). Unlike Ca_V_1.3 WT, the relative nimodipine-sensitive current to the total current was not smaller at night. Moreover, there was no shift toward depolarizing potentials of the peak voltage of the nimodipine-sensitive current-voltage relationship compared to Ca_V_1.3 WT (Figure 2J). Taking into account the lack of change in both the total and nimodipine-sensitive currents in Ca_V_1.3 KO neurons, the results raise the possibility that the loss of Ca_V_1.3 channels might be homeostatically compensated by other LTCCs such as Cav1.2. Thus, the nighttime data are less conclusive and leave open the question of whether Ca_V_1.3 contributes to the night LTCC Ca^2+^ current in SCN neurons.

Ca_V_1.3 KO SCNs thus exhibited a different day vs. night profile for LTCC current magnitude. The daytime change in nimodipine-sensitive current magnitude eliminated the diurnal variation in LTCC current in Ca_V_1.3 KO neurons. Whereas WT neurons had a larger LTCC current during the day (Figure 2D), Ca_V_1.3 KO neurons showed an abrogation of this diurnal difference (Figure 2H). This observation primarily resulted from the decreased daytime nimodipine-sensitive current in Ca_V_1.3 KO compared to WT, which was reduced by about half. The net effect was a loss of the diurnal variation in LTCC current in Ca_V_1.3 KO SCNs.

**Figure 3:**
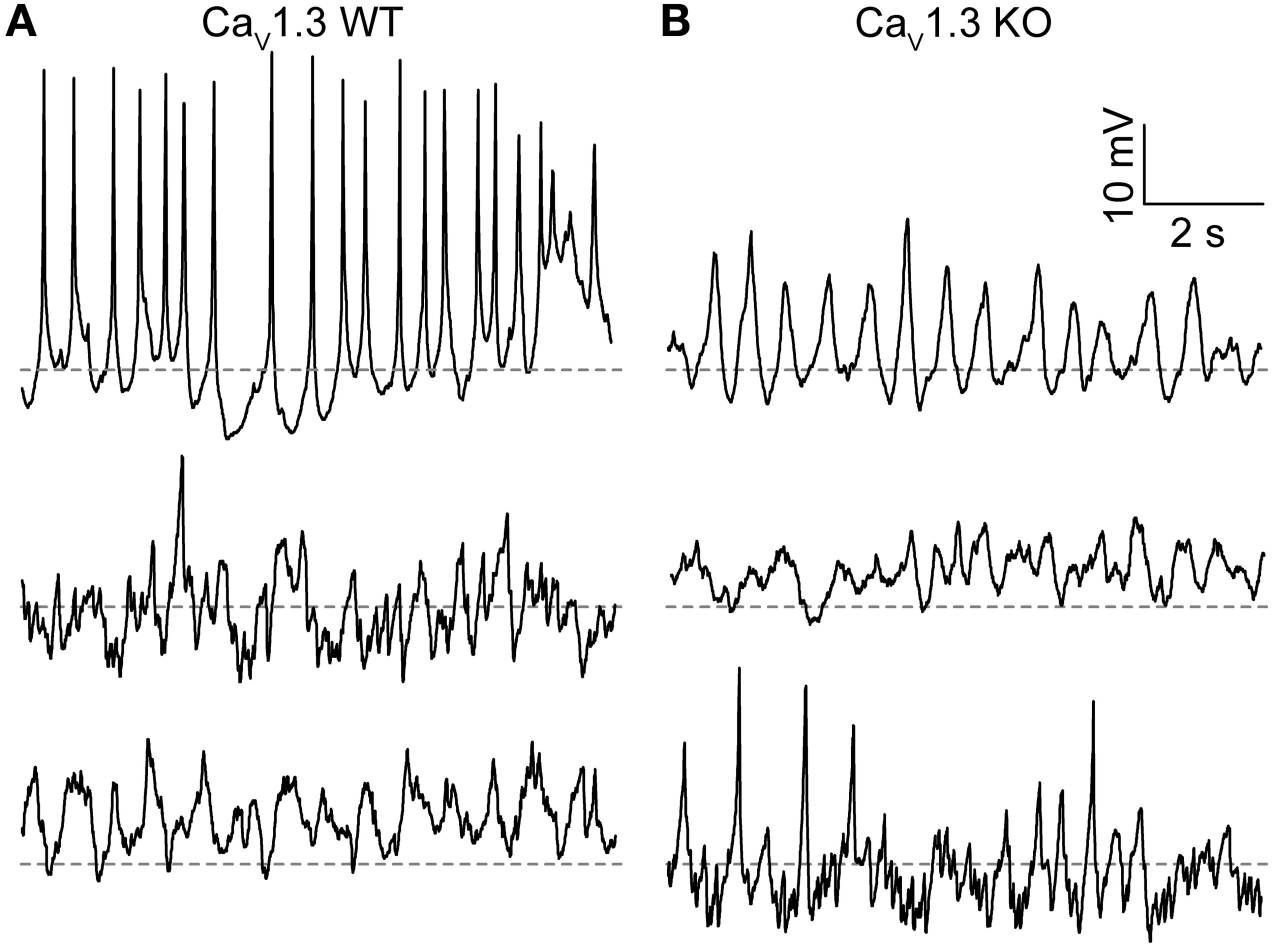
Membrane potential oscillations in Ca_V_1.3 WT and Ca_V_1.3 KO SCN neurons. **(A,B)** Representative current-clamp recordings of membrane potential oscillations from Ca_V_1.3 WT **(A)** and Ca_V_1.3 KO **(B)** neurons recorded during the day. Recordings were made in 1 μM TTX using the same ACSF and 0.9 mM control EGTA intracellular solution used to record BK currents. Dotted lines indicate −40 mV.

Another property of SCN neurons involving LTCCs is the generation of spontaneous subthreshold membrane potential oscillations. During the day, the frequency of these oscillations is higher, and they are eliminated by nimodipine, demonstrating their dependence on LTCC function (Pennartz et al., 2002; Jackson et al., 2004; Huang et al., 2012; McNally et al., 2020). Ca_V_1.3 channels have been proposed to underlie these oscillations due to their activation at subthreshold membrane potentials. Alteration of Ca^2+^-dependent inactivation of Ca_V_1.3 channels is correlated with a decrease in the membrane potential oscillation frequency (Huang et al., 2012). In this study, membrane potential oscillations were revealed in Ca_V_1.3 WT neurons during the day by application of TTX. SCN neurons exhibit a wide range of oscillatory behavior (Figure 3) (Pennartz et al., 2002; Jackson et al., 2004; McNally et al., 2020).

Just over half (56%) of Ca_V_1.3 WT neurons exhibited membrane potential oscillations. Fifteen neurons had spontaneous oscillations, and three neurons had oscillations manifesting with voltage steps from −60 to 0 mV (out of 32 neurons from three slices; Figure 3A). The average resting membrane potential was −44.4 ± 1.8 mV (*n* = 47). Ca_V_1.3 KO neurons also exhibited oscillatory membrane behavior (Figure 3B). Fifty seven percent of Ca_V_1.3 KO neurons exhibited oscillations (six neurons with spontaneous and two neurons with oscillations from voltage-steps, out of 14 neurons total from one slice). The average Ca_V_1.3 KO neuron resting membrane potential was −39.8 ± 2.2 mV (*n* = 14), which was not different from WT (*P* = 0.06, unpaired *t*-test). These results demonstrate that Ca_V_1.3 is not the sole Ca^2+^ channel required to produce spontaneous membrane potential oscillations or regulate resting Ca^2+^-dependent K^+^ conductances in SCN neurons.

### BK Current in Ca_V_1.3 KO SCN Neurons

**Figure 4:**
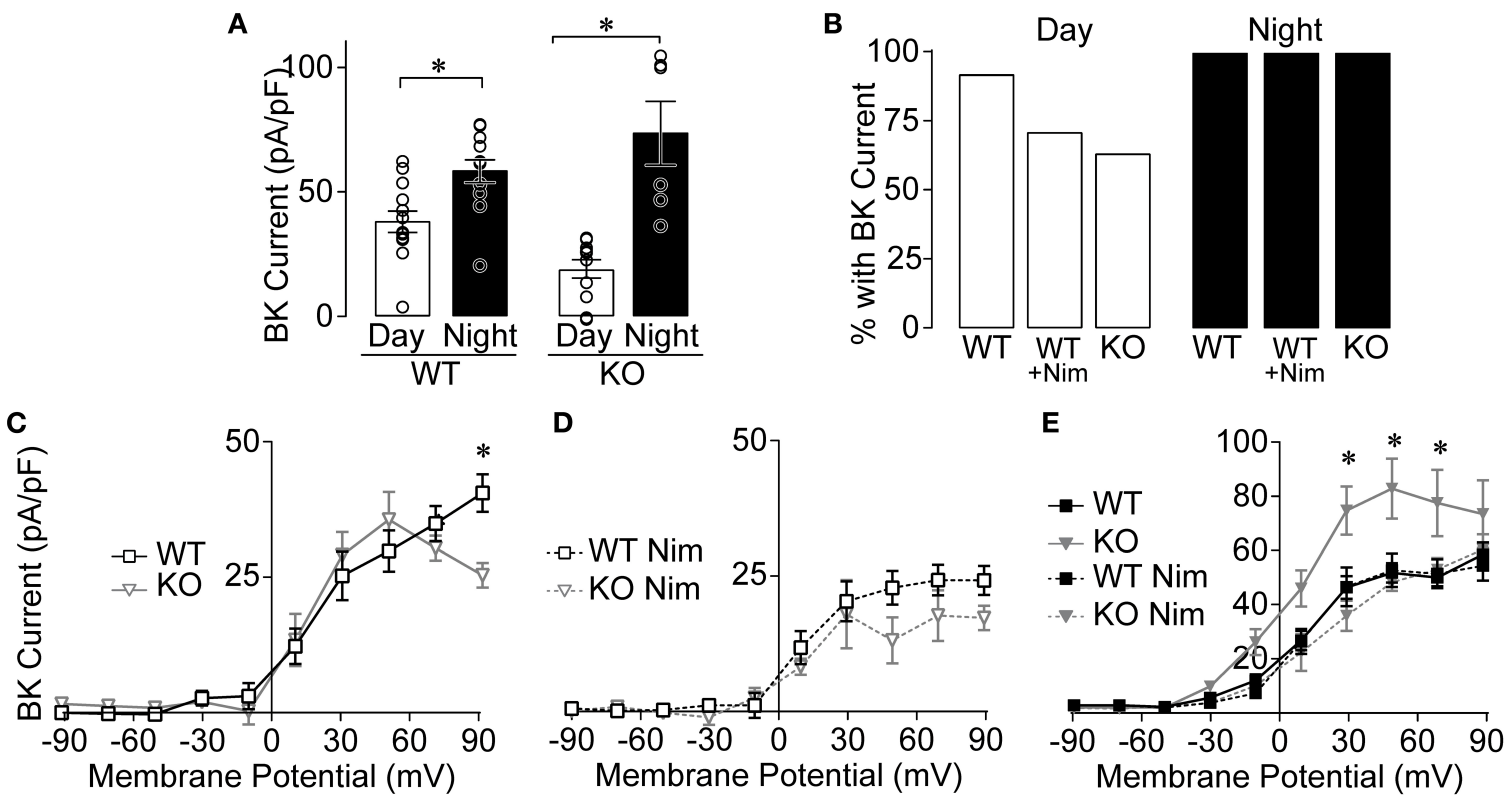
BK currents from Ca_V_1.3 WT and Ca_V_1.3 KO SCN during the day and night. Paxilline-sensitive macroscopic BK currents were recorded as in Figure 1. **(A)** Summary of BK current densities at +90 mV recorded from Ca_V_1.3 WT and Ca_V_1.3 KO neurons during the day and night. BK currents were increased at night for both Ca_V_1.3 WT (*P* = 0.004) and Ca_V_1.3 KO (*P* = 0.007) compared to the day. *P < 0.05, unpaired Welch's *t* tests. **(B)** Percentage of SCN neurons with BK current in Ca_V_1.3 WT and Ca_V_1.3 KO neurons recorded in control conditions or in 10 μM nimodipine (Nim) during the day and night. The number of neurons exhibiting BK currents was not different between Ca_V_1.3 WT and Ca_V_1.3 KO (*P* = 0.14, Fischer's exact test). N's: Ca_V_1.3 WT, 12/13 (cell with BK current/total number recorded); Ca_V_1.3 WT Nim, 10/14; Ca_V_1.3 KO, 7/11 and night: Ca_V_1.3 WT, 12/12; Ca_V_1.3 WT Nim, 13/13; Ca_V_1.3 KO, 6/6. **(C,D)** Current-voltage plot for normalized BK current densities from Ca_V_1.3 WT and Ca_V_1.3 KO neurons in control conditions **(C)** and after application of Nim **(D)** during the day. Ca_V_1.3 WT BK currents were increased at +90 mV compared to Ca_V_1.3 KO in control conditions (*P* = 0.03) but not in Nim (*P* = 0.6). *P < 0.05, Repeated measures ANOVA with Bonferroni's *post hoc* test between WT and KO across −10 to +90 mV. **(E)** Current-voltage plot for BK current densities from Ca_V_1.3 WT and Ca_V_1.3 KO at night, in control and after application of Nim. Ca_V_1.3 KO BK currents were increased at +30 (*P* = 0.007), +50 (*P* = 0.002) and +70 mV (*P* = 0.01) compared to Ca_V_1.3 WT in control conditions at night. N's represent individual cells recorded in (day, night): Ca_V_1.3 WT (13 neurons, six slices; 12 neurons, three slices), Ca_V_1.3 WT Nim (14 neurons, four slices; 13 neurons, four slices), and Ca_V_1.3 KO (11 neurons, four slices; six neurons, two slices), and Ca_V_1.3 KO Nim (three neurons, one slice; three neurons, two slices). Data are mean ± SEM.

Because Ca_V_1.3 makes a clear contribution to the daytime nimodipine-sensitive current (Figure 2), corresponding to the time of day when BK current is more sensitive to nimodipine inhibition (Figure 1F), we investigated the role of this LTCC channel subtype in BK current activation (Figure 4). BK current levels were first quantified at +90 mV, where the largest diurnal difference is routinely quantified (Montgomery and Meredith, 2012; Whitt et al., 2016). We verified that BK current is larger at night in Ca_V_1.3 WT SCN neurons on the mixed strain background (Figure 4A), consistent with previous studies on inbred mouse backgrounds (Pitts et al., 2006; Montgomery and Meredith, 2012; Montgomery et al., 2013). Most Ca_V_1.3 WT neurons had a BK current, both during the day and at night (Figure 4B). In Ca_V_1.3 KO neurons, the day vs. night difference in BK current level also persists in the absence of Ca_V_1.3 channels (Figure 4A). Although the proportion of Ca_V_1.3 KO neurons exhibiting a BK current was lower at 64% during the day, and was similar proportionally to Ca_V_1.3 WT neurons with nimodipine applied, this difference compared to WT was not significant (Figure 4B). At night, all Ca_V_1.3 KO neurons had a BK current. These data show that Ca_V_1.3 is not absolutely required to activate BK currents in most neurons, or for the overall circadian difference in BK current magnitude in the SCN.

In whole-cell recordings, multiple Ca^2+^ channel subtypes can contribute to BK current activation. Although Ca_V_1.3 KO neurons with BK currents do not require Ca_V_1.3 channels, these channels may still contribute to BK current activation in combination with other LTCC subtypes (such as Ca_V_1.2). This possibility was addressed by examining the current-voltage relationships for Ca_V_1.3 WT and KO BK currents. In this comparison for daytime SCN neurons, there was little difference in BK current levels across the voltage range, except at the highest voltage (Figure 4C). Although this reduction could suggest contribution from Ca_V_1.3 channels to BK current activation, the peak Ca^2+^ influx due to Ca_V_1.3 channels occurs around −20 to −10 mV. For BK channels activated directly by Ca_V_1.3 Ca^2+^ currents, the largest relative BK current activation is thus observed between −10 and 0 mV (Vivas et al., 2017; Plante et al., 2021). Lack of a difference in BK current observed at these voltages suggests that Ca_V_1.3 channels may not make a notable contribution to BK current activation in SCN neurons during the day.

When BK channels are coupled to the other neuronal L-type Ca^2+^ channel subtype, Ca_V_1.2, the BK current activation profile is shifted to higher voltages (+30 mV) (Berkefeld et al., 2006; Plante et al., 2021). In the Ca_V_1.3 KO, the contribution of Ca_V_1.2 channels to BK current activation can be indirectly inferred by applying nimodipine. Inhibiting the remaining LTCC current in Ca_V_1.3 KO neurons reduced BK current levels (at +50 mV, *P* = 0.006, two-way repeated measures ANOVA with Bonferroni's *post hoc* test) (Figure 4C vs. D), suggesting that it can be attributed to Ca_V_1.2 channels. However, in the presence of nimodipine, BK current levels were not significantly different between Ca_V_1.3 WT and KO neurons (Figure 4D). The reduction in BK current to similar levels with nimodipine in both genotypes indirectly suggests that there is little compensatory upregulation of Ca_V_1.2 that affects BK current during the day.

At night, there was a paradoxical increase in BK current in Ca_V_1.3 KO neurons compared to Ca_V_1.3 WT (Figure 4E). However, in this case, the increase likely comes from compensatory upregulation of Ca_V_1.2, since nimodipine reduces BK current back to WT levels. This result precludes formulating conclusions about the contribution of Ca_V_1.3 to BK current activation in nighttime neurons in this study. Yet because nimodipine normally does not significantly decrease the nighttime BK current level in WT neurons in this (Figure 1F) and prior studies (Whitt et al., 2018), it is reasonable to conclude that there is no major role for Ca_V_1.3 channels in BK current activation at night.

## Discussion

Ca_V_1.3 has been proposed to play a role in circadian excitability based on its expression and functional modulation in SCN neurons (Huang et al., 2012; Kim et al., 2015; Cheng et al., 2018). Here we show that Ca_V_1.3 channels contribute to the daytime LTCC current in SCN neurons. Their contribution may account for up to half of the LTCC current during the day, providing part of the basis for the diurnal difference in Ca^2+^ current levels. In contrast, it is not clear whether Ca_V_1.3 contributes to daytime membrane potential oscillations or contributes to the nighttime LTCC current. Spontaneous Ca^2+^ oscillations are dependent on the function of LTCCs (Pennartz et al., 2002; Jackson et al., 2004; Huang et al., 2012; McNally et al., 2020). Although Ca_V_1.3 has been proposed to be important for generating these oscillations (Huang et al., 2012; Comunanza et al., 2014), the presence of membrane oscillations in Ca_V_1.3 KO neurons posits that other Ca^2+^ channels are also competent to generate them. Moreover, at night where there is no reduction of the nimodipine-sensitive Ca^2+^ current in Ca_V_1.3 KO cells and no change in the total Ca^2+^ current, it suggests that Ca_V_1.3 does not make a significant contribution to the nighttime LTCC current in SCN neurons. However, the data do not completely rule out the possibility of a compensatory upregulation of another LTCC channel subtype, such as Ca_V_1.2, that is sufficient to maintain the nighttime Ca^2+^ current in Ca_V_1.3 KO SCNs at a similar level to that observed for Ca_V_1.3 WT. This compensation is further suggested by the increase in BK current observed in Ca_V_1.3 KO neurons at night. In other tissues, compensation has been shown to occur *via* upregulation of Ca_V_1.2 in the absence of Ca_V_1.3 function (Namkung et al., 2001; Zhang et al., 2005; Marcantoni et al., 2010; Jurkovičová-Tarabová et al., 2012; Poetschke et al., 2015).

BK channels are Ca^2+^-activated, and LTCCs are a major source of Ca^2+^ required for their activation during the day in SCN neurons (Whitt et al., 2018). Several factors motivated the specific investigation of Ca_V_1.3 channels in BK current activation. In several types of excitable cells, in heterologous cells, and across diverse animal species, BK and Ca_V_1.3 channels have been shown to functionally couple (Grunnet and Kaufmann, 2004; Berkefeld et al., 2006; Marcantoni et al., 2010; Bellono et al., 2017; Vivas et al., 2017; Plante et al., 2021). In this study, we found that while the contribution of Ca_V_1.3 channels to the daytime LTCC current is significant, the effect on BK current is more limited. First, the day vs. night difference in BK current levels that is critical for maintaining proper circadian rhythm in SCN activity is still expressed in the Ca_V_1.3 KO. This stands in contrast to the LTCC current recorded from Ca_V_1.3 KO neurons, which does not show a diurnal difference in levels. Second, many SCN neurons still possess detectable BK currents and retain mostly normal BK current activation in the Ca_V_1.3 KO. Lastly, in prior behavioral studies, daily patterns of locomotor activity were shown to be essentially normal in Ca_V_1.3 KO mice on a regular light:dark cycle (Busquet et al., 2010). If Ca_V_1.3 were a fundamental source of Ca^2+^ for BK channel activation, some disruption of locomotor activity might be expected, along the lines of the alterations in behavioral rhythms observed due to loss of BK channel function (Meredith et al., 2006). Taken together, these data suggest that Ca_V_1.3 channels are not the predominant LTCCs contributing to the net steady-state BK current levels under these recording conditions.

Yet the contribution of Ca_V_1.3 channels could be more subtle or underestimated. BK–Ca_V_1.3 channel complexes expressed in heterologous cells were sensitive to low concentrations of BAPTA (0.1 mM) (Vivas et al., 2017), while the SCN BK currents in this study were resistant to higher concentrations of BAPTA (5 mM). This raises the possibility that the predominant BK—LTCC complex in SCN neurons is more tightly coupled than BK–Ca_V_1.3 channel complexes, making their contribution harder to assess. There were also some minor changes in BK currents from daytime SCN neurons, such as a trend toward fewer neurons with detectable BK currents and a drop-off in the BK current at the peak voltage tested compared to WT. The lack of significance in this data could result from the variability in the Ca^2+^ currents recorded between SCN neurons (Whitt et al., 2018; McNally et al., 2020). Moreover, it is possible that the major contribution for Ca_V_1.3 channels could be in the smaller population of neurons that had no BK currents in Ca_V_1.3 KO neurons. Alternatively, the changes in BK current observed at +90 mV in the Ca_V_1.3 KO could reflect an indirect role for the channel, such as in the gating of RyR-mediated Ca^2+^ release. A small portion of daytime SCN BK current has been previously reported to be sensitive to dantrolene (Whitt et al., 2018), but the pathway mediating the opening of RyRs is not currently understood in SCN neurons (Harvey et al., 2020). Lastly, Ca_V_1.3 is less sensitive nimodipine than Ca_V_1.2 (Xu and Lipscombe, 2001), raising the possibility that a small contribution to BK current could be lost within the variability of Ca^2+^ currents from cell to cell.

The data in this study indirectly suggest Ca_V_1.2 as a more significant LTCC for providing the Ca^2+^ influx that activates BK channels in the daytime SCN. BK current activation is preserved to a large extent when Ca_V_1.3 channels are absent, and a portion of that current is sensitive to nimodipine. BK channels are also well-described to couple to Ca_V_1.2 channels in a variety of cell types, including in neurons and heterologous cells (Berkefeld et al., 2006, 2010; Berkefeld and Fakler, 2008; Hou et al., 2016; Plante et al., 2021). Besides Ca_V_1.3, Ca_V_1.2 is likely the relevant LTCC subtype to consider in SCN neurons, since Ca_V_1.1 is restricted to skeletal muscle and Ca_V_1.4 to retina (Lipscombe et al., 2004; Lee et al., 2015).

A factor complicating some interpretations in this study include the issue of LTCC compensation in the Ca_V_1.3 KO. Substantia nigra and lateral superior olive neurons, as well as adrenal chromaffin cells, exhibit upregulation of other Ca^2+^ currents in Ca_V_1.3 KOs (Marcantoni et al., 2010; Jurkovičová-Tarabová et al., 2012; Poetschke et al., 2015). A distinct Ca_V_1.3 KO mouse line also exhibited compensatory upregulation of Ca_V_1.2 channels in heart tissues and pancreatic β-cells (Namkung et al., 2001; Zhang et al., 2005), suggesting Ca_V_1.3 and Ca_V_1.2 have a connected expression relationship. In this study, the increased BK current at night in Ca_V_1.3 KO neurons comes from LTCC compensation, since it is nimodipine sensitive. However, the total and nimodipine-sensitive Ca^2+^ currents did not increase compared to WT, as expected if there were compensation. This observation leaves open the question of whether LTCC compensation actually obscured a decrease in the Ca_V_1.3 KO LTCC current at night. While this issue makes it difficult to assess the role for Ca_V_1.3 in the nighttime Ca^2+^ current, it is unlikely to affect the conclusion that it does not play a role in BK current activation at night. Interestingly, the increase in BK current at night suggests that when extra LTCC channels are made, they can aberrantly couple to BK channels at the wrong time of day. Since it is not yet known how BK channels change their functional Ca^2+^ channel associations over the circadian cycle in SCN (Whitt et al., 2018; Harvey et al., 2020), the Ca_V_1.3 KO model may provide a new context to test mechanisms for BK–Ca_V_ coupling.

## Data Availability Statement

The raw data supporting the conclusions of this article will be made available by the authors, without undue reservation.

## Ethics Statement

The animal study was reviewed and approved by the Institutional Animal Care and Use Committee at the University of Maryland, Baltimore.

## Author Contributions

BM performed the experiments. BM, AP, and AM analyzed the data. AM wrote the manuscript. All authors contributed to the article and approved the submitted version.

## Conflict of Interest

The authors declare that the research was conducted in the absence of any commercial or financial relationships that could be construed as a potential conflict of interest.

## Publisher's Note

All claims expressed in this article are solely those of the authors and do not necessarily represent those of their affiliated organizations, or those of the publisher, the editors and the reviewers. Any product that may be evaluated in this article, or claim that may be made by its manufacturer, is not guaranteed or endorsed by the publisher.
